# Case Report: A young man with frontal traumatic sinus pericranii

**DOI:** 10.3389/fsurg.2024.1479233

**Published:** 2024-12-03

**Authors:** Zihao Zhang, Qingpei Hao, Ruirui Luan, Guangbiao Qin, Ruen Liu

**Affiliations:** ^1^ Department of Neurosurgery, Peking University People’s Hospital, Beijing, China; ^2^ Department of Pathology, Peking University Cancer Hospital, Beijing, China

**Keywords:** sinus pericranii, venous anomaly, trauma, treatment, management

## Abstract

Sinus pericranii is a rare venous anomaly characterized by abnormal communication between intracranial and subperiosteal veins, and reports of trauma-induced sinus pericranii are even rarer. Herein, we report a case of delayed-onset sinus pericranii resulting from a traumatic injury to the left side of the midline of the forehead sustained in early childhood. The anomaly was successfully resected via a coronal incision within the hairline, followed by meticulous bone wax sealing. In this paper, we aim to provide details on the diagnosis and surgical techniques of trauma-induced sinus pericranii, contributing valuable insights for the management of such rare condition.

## 1 Introduction

Sinus pericranii (SP) is predominantly a congenital condition, but it can also develop secondary to head trauma. Trauma-induced SP is exceptionally rare, with only several documented cases in the literature (1, 2). While the diagnosis of SP is generally straightforward, it is crucial not to overlook potential underlying conditions such as craniosynostosis, cephalocele, arteriovenous malformation (AVM), venous angioma, cavernous angioma, hydrocephalus and other congenital anomalies, which can lead to SP (3–11). This venous anomaly may initially be mistaken for a simple scalp swelling and improperly treated, necessitating increased vigilance. Moreover, rapid enlargement of SP within a short period demands even greater vigilance (4). Although the prognosis for SP patients is relatively favorable, achieving aesthetically pleasing and precise management, especially for frontal SP, remains challenging. This case report aims to present our experience in managing trauma-induced frontal SP and to review the existing literature, providing valuable insights for the diagnosis and treatment of this rare condition.

## 2 Case report

### 2.1 Patient information

**Figure 1:**
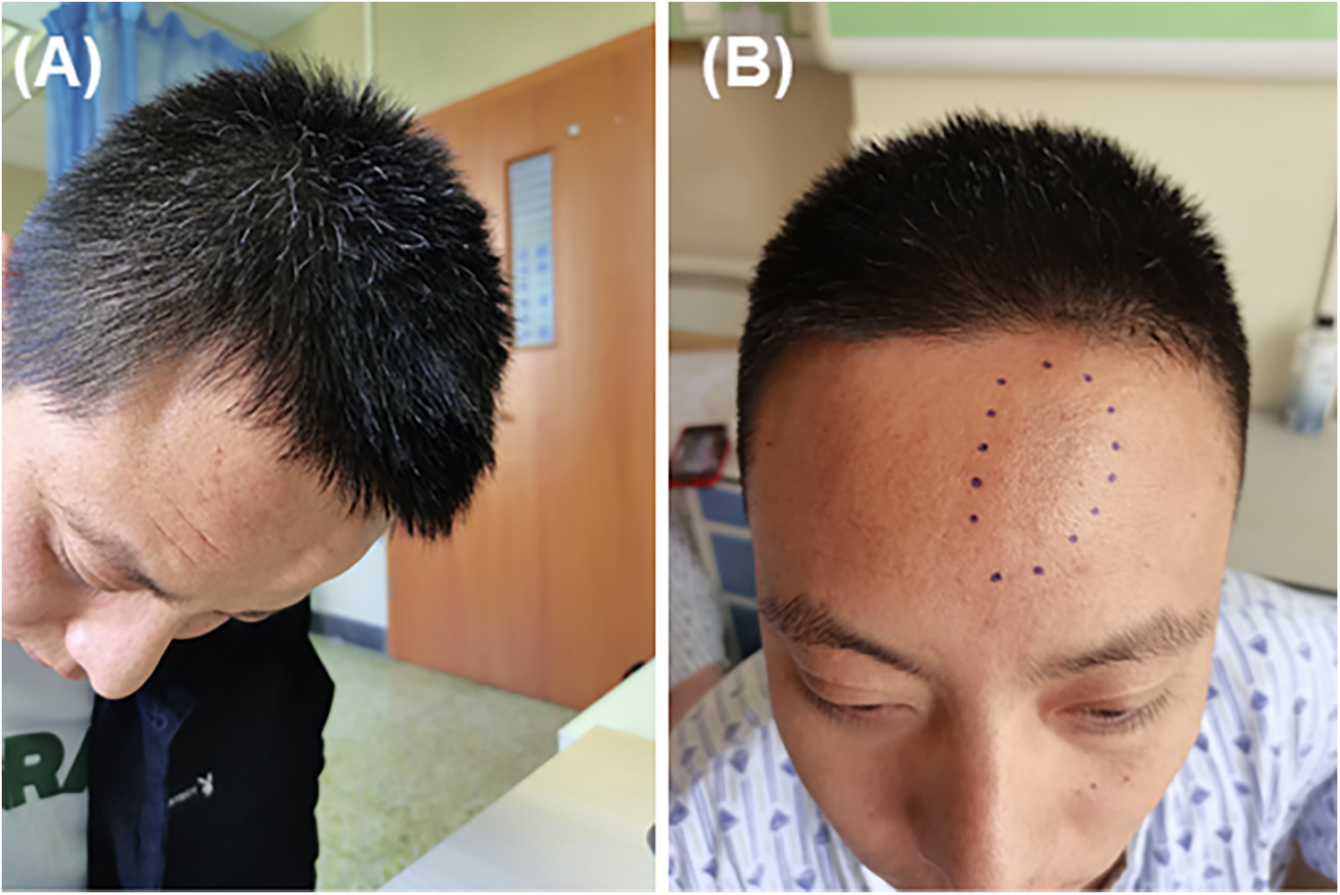
The mass was located slightly to the left of the midline in the frontal region. **(A)** The mass enlarged when the head was lowered. **(B)** The mass was not noticeable when standing or sitting.

A 31-year-old man presented with a six-year history of a nonpulsatile swelling in the frontal region. The swelling was asymptomatic and intermittent, persisting for a few days each time before regressing without treatment. It measured 4 cm by 2 cm in diameter when standing, enlarged in the head-down position or with Valsalva maneuver, and had a fluctuant consistency upon palpation (Figures 1A,B). The patient recalled a injury around frontal midline in childhood that was not treated. There was no history of previous intracranial surgery or infection.

**Figure 2:**
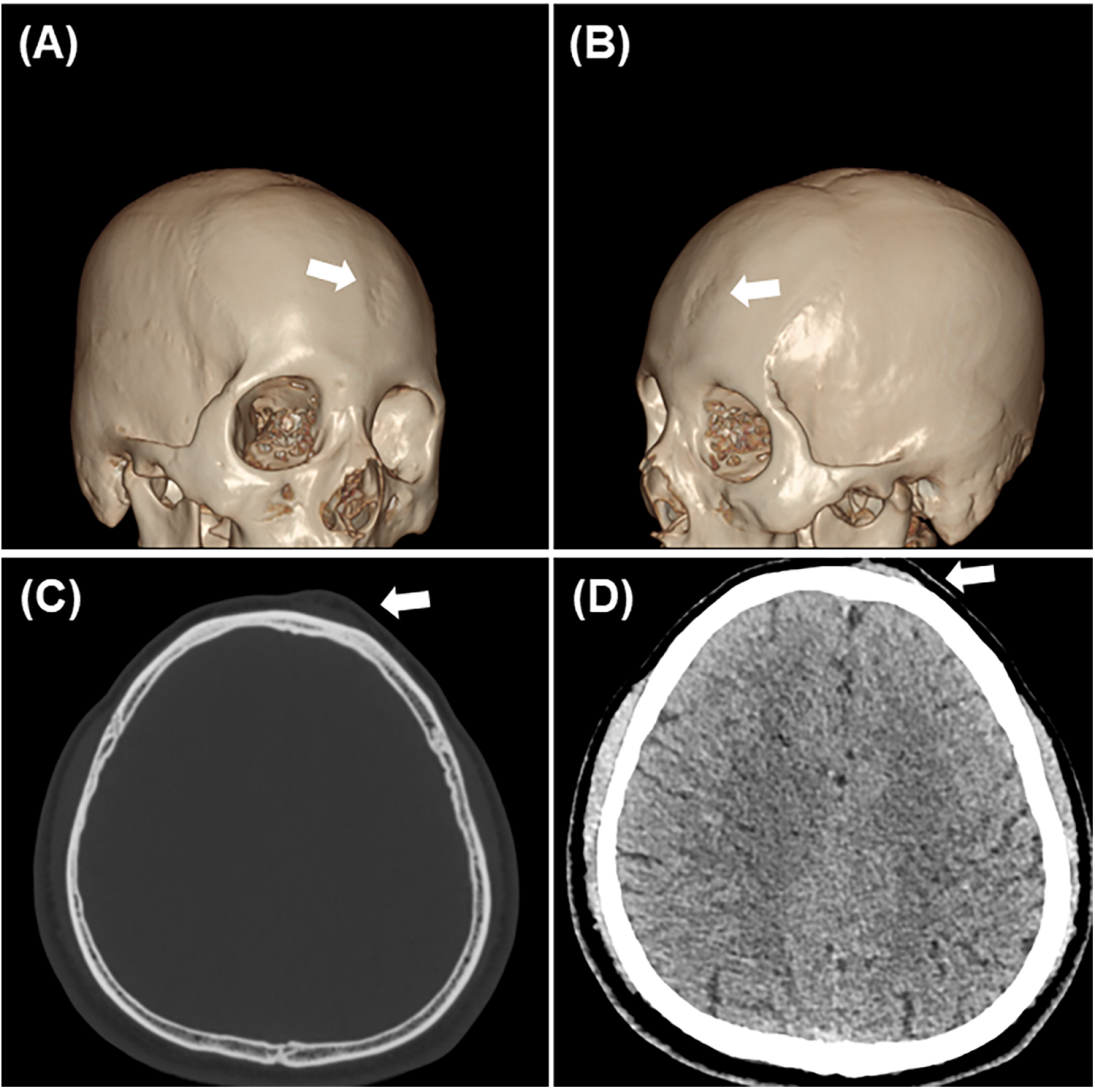
Imaging data. **(A,B)** Three-dimensional reconstruction indicated that the skull was rough and irregular, with numerous small holes on the surface (white arrow). **(C,D)** Axial imaging revealed localized bone thinning and a subcutaneous mass(white arrow).

Three-dimensional Computed Tomography (CT) revealed a thin-walled region of the skull approximately 3 cm in diameter with several small holes and irregular, roughened bone, showing several small transosseous vessels extending through the surface of the skull (Figures 2A,B). Axial CT imaging further demonstrated localized bone thinning and a subcutaneous mass in the same region (Figures 2C,D). Based on these findings, a diagnosis of sinus pericranii was made. Angiography revealed no obvious thrombi, dural sinus abnormalities, or other vascular malformations, indicating that excision of the sinus pericranii was not affecting intracranial venous perfusion.

### 2.2 Neurosurgical procedure

**Figure 3:**
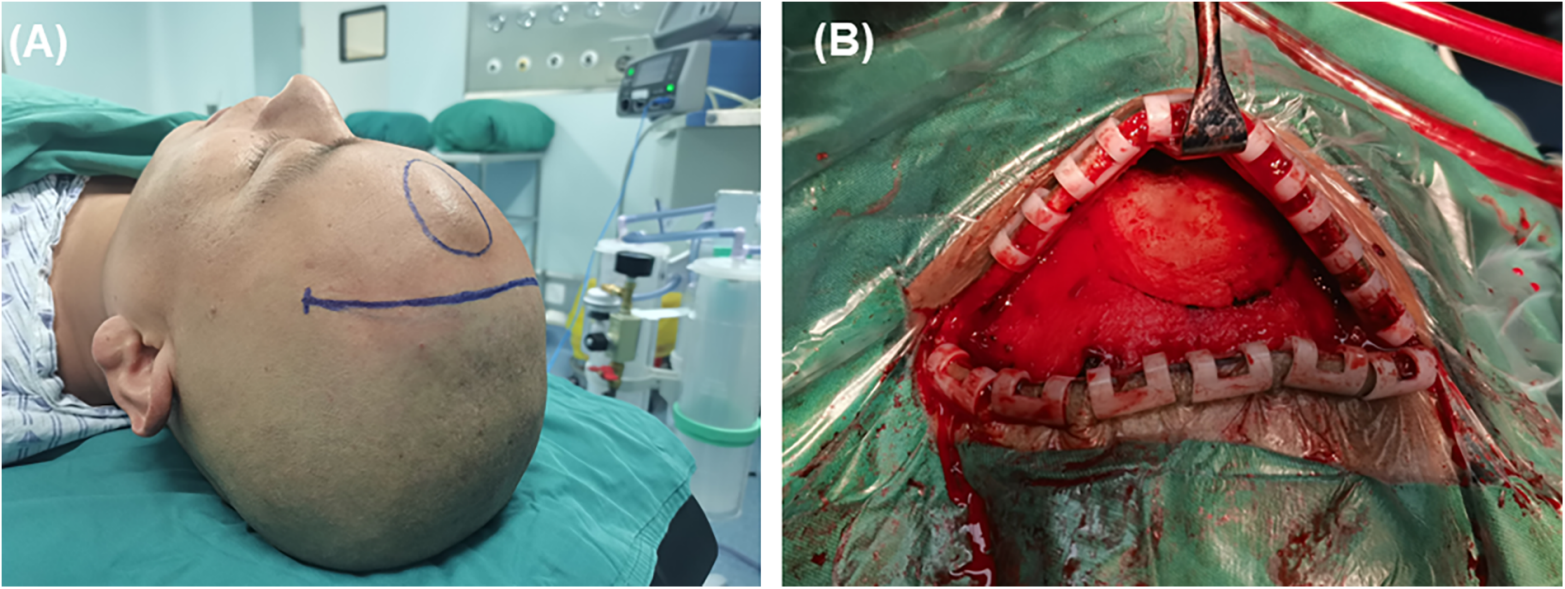
Choice of surgical incision and surgical findings. **(A)** The patient was placed in a supine position, and to avoid scarring on the forehead, a coronal incision within the hairline was chosen. **(B)** Bone wax was used to thoroughly seal the small holes on the surface of the skull to prevent recurrence.

After obtaining written informed consent, a curvilinear incision was made adjacent to the hairline (Figure 3A). The skin flap was carefully dissected from the underlying periosteal membrane, revealing the fluctuant mass beneath the galea. Numerous vascular channels, approximately 1 mm in diameter, were noted on the depressed skull, with venous blood gushing out from these holes. The vascular mass was meticulously coagulated and dissected from the skull surface. Complete excision of the mass was achieved, and the skull holes were sealed with bone wax (Figure 3B). The incision was then sutured, ensuring optimal cosmetic and functional outcomes. Within 24 h post-operation, there was no bleeding, pneumatosis, or effusion observed. The patient was successfully discharged.

### 2.3 Histopathological examination

**Figure 4:**
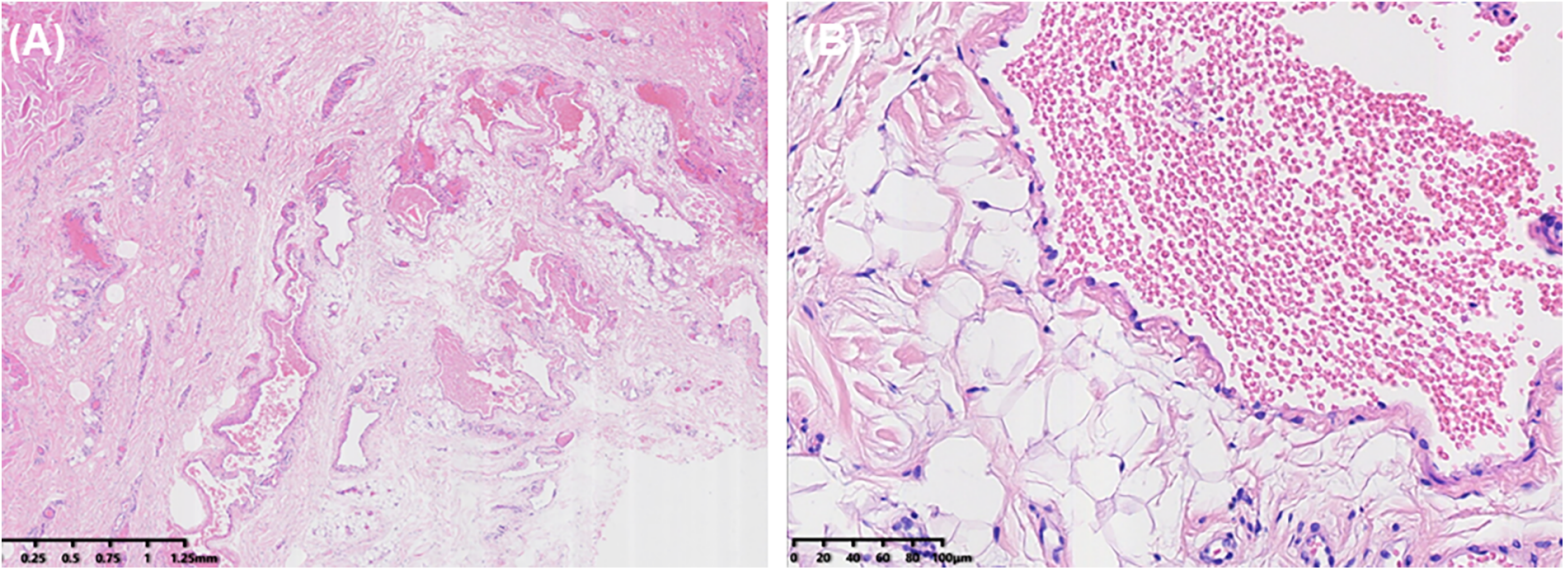
Histopathological examination. **(A)** Within the fibrous tissue, multiple thin-walled blood vessels were observed, some merging to form a sieve-like pattern. **(B)** The vessel walls were thin, lined with a single layer of endothelial cells, and lacked both muscular and elastic layers.

Histopathological examination revealed numerous thin-walled blood vessels within the fibrous tissue, some of which merged and overlapped to form a sieve-like pattern. Most of the lumens are irregular, lined with a single layer of endothelial cells, and lacked both muscular and elastic layers. Some vessel walls were incomplete and contained red blood cells, indicating congestion and hemorrhage (Figures 4A,B). These findings are consistent with the clinical diagnosis of sinus pericranii.

### 2.4 Follow-up and outcomes

The young man recovered well, with no recurrence at the 1-month outpatient follow-up. At the 3-month follow-up, he remained free of recurrence, headache, or local pain.

## 3 Discussion

G. Stromeyer (12) first introduced the term “Sinus pericranii” in 1,850 to describe a blood-filled reservoir on the skull bones that communicates with the dura mater sinuses via the diploic veins. Although the exact incidence of SP is not clearly documented, it is known to be a rare vascular malformation, typically presenting as a sporadic scalp mass, with most cases being congenital (9, 13–16). Traumatic frontal SP is an even rarer, with only a few dozen cases reported (1, 2, 17). The limited data hinders a comprehensive understanding of SP, and no established treatment protocol exists. A significant proportion of cases are misdiagnosed as simple scalp masses and excised, which poses substantial risks (18, 19).

The hidden risks are primarily due to two main factors: First, SP is often classified into two types: (a) Secondary: often caused by trauma, hydrocephalus, intracranial hypertension; (b) Congenital: most common in craniosynostosis and frequently accompanied by various venous malformations and developmental anomalies (6–9). Treating SP as a simple scalp mass, regardless of its type, obscures the underlying condition and overlooks the true problem. Second, SP can be categorized as dominant (if the majority of venous flow communicates through the SP) or accessory (if only a portion of the intracranial venous flow communicates through the SP): For accessory SP, treatments such as surgical excision or vascular intervention are feasible; However, for dominant SP, excision as a simple scalp mass can lead to severe complications such as cerebral edema (20). Therefore, early and precise diagnosis of SP is crucial for determining the appropriate treatment, reducing complications, and optimizing therapeutic outcomes.

When diagnosing SP, imaging studies are more critical than physical examinations and medical history. Three-dimensional CT scans can clearly reveal any thinning, defects, or holes in the skull beneath the mass and assess for craniosynostosis (7, 11, 21, 22). CT with contrast or Magnetic Resonance Venography (MRV) can demonstrate the presence of extracranial blood vessels within the mass. If necessary, Digital Subtraction Angiography (DSA) can be used to confirm the communication between extracranial veins and intracranial venous sinuses or dural venous lakes (10, 23, 24). By integrating these imaging findings with the patient's medical history and symptoms, a diagnosis of SP can be established (3, 4, 25–27). However, not all imaging studies are necessary in every case.

In our case, the patient, a 31-year-old man, presented with an asymptomatic mass. A three-dimensional CT scan was performed to identify skull abnormalities and exclude hydrocephalus, intracranial lesions and hypertension. The patient had a clear history of frontal trauma at the site of the mass during childhood. Angiography also ruled out intracranial vascular malformations and the type of dominant SP. Based on these findings, a diagnosis of SP was confirmed, and surgical excision was deemed appropriate.

The treatment of SP is challenging due to its low incidence, which results in limited reports and a lack of established diagnostic or treatment guidelines. Currently, endovascular embolization and surgical resection are the two main treatment methods (4, 10, 14, 20, 23, 28). After excluding secondary factors such as malformations and hydrocephalus, surgical resection can be performed. Given the reports of recurrence following resection, we reviewed the relevant literature and combined it with our experiences to propose the following measures for preventing postoperative recurrence: For the initial treatment of typical SP, it is essential to coagulate and remove all abnormal vessels and seal all holes with bone wax; For patients with high-risk factors for recurrence, such as an unusually large number of emissary veins or rich circulation between intra- and extracranial venous systems, we recommend coagulating and severing all veins at their point of departure from the sinus; In case of large bone defects, it is advisable to use autologous bone grafts or artificial bone graft materials, such as acrylic resin, to seal the defects and the surrounding thinned skull, followed by covering the lesion with periosteum; For patients with concurrent conditions like raised intracranial pressure, hydrocephalus, cerebral vascular malformations, or increased intracranial venous pressure, it is crucial to first address these primary conditions before proceeding with the resection of SP (1, 3–9, 14, 15, 21, 22, 25–34).

## 4 Conclusion

In this case, we describe a patient with traumatic frontal SP. The choice of incision site, complete resection, and prevention of recurrence are crucial for both cosmetic outcomes and effective treatment of frontal SP. Pathological examination is essential for confirming diagnosis. Through this rare case and a review of the existing literature, we aim to provide valuable insights for the diagnosis and management of SP.

## Data Availability

The original contributions presented in the study are included in the article/Supplementary Material, further inquiries can be directed to the corresponding authors.
